# SARS CoV-2 Delta variant exhibits enhanced infectivity and a minor decrease in neutralization sensitivity to convalescent or post-vaccination sera

**DOI:** 10.1016/j.isci.2021.103467

**Published:** 2021-11-15

**Authors:** Alona Kuzmina, Seraj Wattad, Yara Khalaila, Aner Ottolenghi, Benyamin Rosental, Stanislav Engel, Elli Rosenberg, Ran Taube

**Affiliations:** 1 The Shraga Segal Department of Microbiology Immunology and Genetics, Faculty of Health Sciences, Ben-Gurion University of the Negev, Beer Sheva, Israel; 2 Department of Clinical Biochemistry and Pharmacology Faculty of Health Sciences, Ben-Gurion University of the Negev, Beer Sheva, Israel; 3 Soroka Medical Center, Beer Sheva, Israel; 4 Regenerative Medicine and Stem Cell Research Center, Ben Gurion University of the Negev, Beer Sheva, Israel

**Keywords:** Immunology, Virology

## Abstract

Since their identification, severe acute respiratory syndrome coronavirus 2 (SARS-CoV-2) Kappa and Delta have rapidly spread to become globally dominant. However, their infectivity and sensitivity to administered vaccines have not been documented. We monitored the neutralization potential of convalescent or BNT162b2 post-vaccination sera against Kappa and Delta SARS-CoV-2 pseudoviruses. We show that both variants were successfully neutralized by convalescent and post-vaccination sera, exhibiting a mild decrease in their neutralization sensitivity. Of the two variants, Delta presented enhanced infectivity levels compared with Kappa or wild-type SARS-CoV-2. Nevertheless, both variants were not as infectious or resistant to post-vaccination sera as the Beta variant of concern. Interestingly, the Delta plus variant (AY.1/B.1.617.2.1) exhibited high resistance to post-vaccination sera, similar to that of the Beta SARS-CoV-2. However, its infectivity levels were close to those of wild-type SARS-CoV-2. These results account for the worldwide prevalence of Delta variant of concern and confirm the efficacy of the BNT162b2 vaccine against circulating other Delta variants.

## Highlights

• Pfizer vaccine efficiently neutralizes Kappa and Delta SARS-CoV-2 variants
• The Delta variant exhibits high infectivity levels relative to wild-type SARS-CoV-2
• Beta and Delta plus variants exhibit increased neutralization resistance to vaccine

## Graphical abstract

**Figure:**
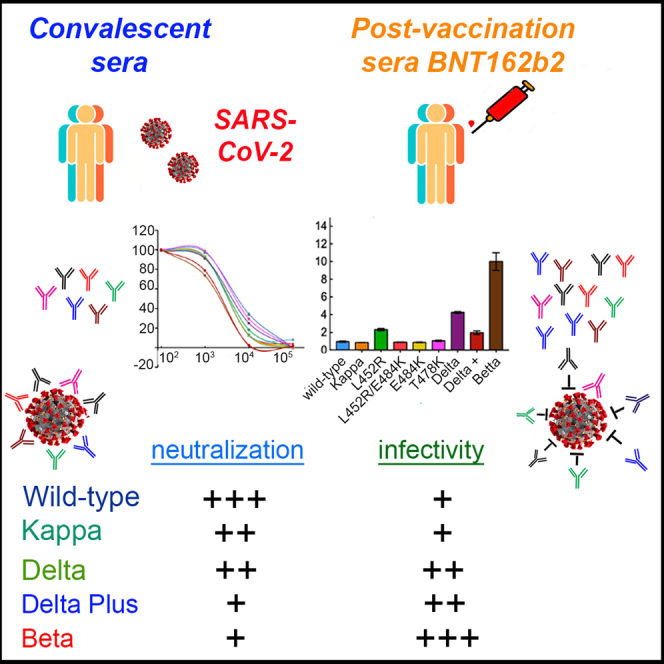

## Introduction

In late 2019, an emergent betacoronavirus, severe acute respiratory syndrome coronavirus 2 (SARS-CoV-2) was identified as the cause for severe respiratory disease in humans. One year into this outbreak, coronavirus disease 2019 (COVID-19) is a global pandemic forcing most countries to adopt a lockdown mode, causing economic burden and human suffering with more than 178 million cases and 3.8 million deaths (Zhou et al., 2020; Zhu et al., 2020). SARS-CoV-2 infection of its target cells occurs via the spike glycoprotein, S, a trimeric class I fusion transmembrane glycoprotein with S1 and S2 subunits that are non-covalently associated. Within S1, the receptor binding domain (RBD; residues 331–528) attaches the human angiotensin-converting enzyme 2 (ACE2) receptor to mediate viral cell attachment and entry (Bestle et al., 2020; Hoffmann et al., 2020a, 2020b; Johnson et al., 2021; Letko et al., 2020; Papa et al., 2021; Walls et al., 2020; Wrapp et al., 2020; Zang et al., 2020). RBD is the main target for neutralizing antibodies (nAbs), which are elicited following infection or vaccination and can inhibit viral entry. Key mutations within spike improve the affinity of the virus to ACE2, resulting in enhanced transmissibility. Other mutations within RBD and the N-terminal domain of spike also promote immune escape from nAb (Alsoussi et al., 2020; Brouwer et al., 2020; Gaebler et al., 2021; Group et al., 2020; Klasse and Sattentau, 2002; Plotkin, 2010; Rogers et al., 2020; Wu et al., 2020). Current available vaccines, BNT162b2 and mRNA-1273, have been proved to be highly efficient at limiting COVID-19 disease progression (Anderson et al., 2020; Baden et al., 2021; Krammer, 2020; Muik et al., 2021; Polack et al., 2020; Walsh et al., 2020). However, rapid viral spread, extensive genetic viral evolution, and incomplete vaccination have potentially led to the emergence of viral variants that escape nAb efficacy. Widespread variants of concern include the Alpha-B.1.1.7 that was first identified in the United Kingdom (Korber et al., 2020; Meng et al., 2021), Beta B.1.351 that was isolated in South Africa (Hou et al., 2020; Plante et al., 2021), and Gamma-P1 lineage isolated in Brazil. Key mutations within spike of Alpha-B.1.1.7 include the dominant D614G mutation, which enhances viral transmission (Hou et al., 2020; Korber et al., 2020; Plante et al., 2021; Zhou et al., 2021); deletions; and missense mutations within spike. Importantly, an N501Y mutation increases the affinity of spike to ACE2 and the P681H mutation within the furin cleavage site (FCS) increases viral transmissibility. Beta-B.1.351 variant also carries RBD substitutions, including K417N, E484K, and N501Y. Among these, E484K and K417N are key for antibody escape and reduced sensitivity to administrated vaccines (Greaney et al., 2021; Kuzmina et al., 2021; Starr et al., 2020; Xie et al., 2021) (Baum et al., 2020; Ku et al., 2021; Weisblum et al., 2020; Wibmer et al., 2021) (Andreano et al., 2021; Xie et al., 2021) (Giandhari et al., 2020). Variants of interest include the lota-B.1.526 variant in New York and the B.1.429 California variant, each exhibiting unique mutations within their spike (Annavajhala et al., 2021; Candido et al., 2020; Sabino et al., 2021; Tegally et al., 2021; West et al., 2021) (Deng et al., 2021; Shen et al., 2021). Recently, a new variant of interest, B.1.617, was identified in India and has rapidly spread, initially in the United Kingdom and thereafter worldwide, where it became dominant. B.1.617 consists of several lineages, where Kappa-B.1.617.1 variant of interest carries L452R, E484Q, and P681H spike mutations, whereas Delta-B.1.617.2 linage carries L452R, T478K, and P681R unique mutations. However, the efficacy of current administered vaccines against Kappa-B.1.617.1 and Delta-B.1.617.2 lineages has not been fully investigated, and the reason for its rapid spread has not been determined. Recent reports demonstrated that the Covishield-AstraZeneca vaccine induced nAb response against the Kappa-B.1.617.1, with a 2-fold reduction in antibody titer. Despite this, the authors conclude that vaccine-induced immunity still limits the severity of COVID-19 and mortality in vaccinated individuals (Di Giacomo et al., 2021; Yadav et al., 2021). Herein, we used pseudoviruses to monitor the efficacy of convalescent or post-vaccination sera to neutralize Kappa-B.1.617.1 SARS-CoV-2 or Delta-B.1.617.2 variants. We showed that both B.1.617.1 and B.1.617.2 were successfully neutralized by convalescent or post-vaccination sera, exhibiting only a mild (about 2-fold) decrease in their sensitivity. Interestingly, Delta-B.1.617.2 exhibited 4.2-fold enhancement in viral infectivity relative to wild-type SARS CoV-2 (Wuhan-Hu-1isolate), or its Kappa-B.1.617.1 lineage, which showed similar levels of infectivity as wild-type pseudovirus. We also monitored the effects of single or combined RBD mutations that appear in the Kappa or Delta variants. We show that the single L452R spike mutation, which is shared by both B.1.617.1 and B.1.617.2, is critical for the unique phenotype of B.1.617.2. L452R-pseudoviruses exhibited a 2.14-fold reduction in post-vaccination neutralization potential against post-vaccination sera relative to wild-type SARS-CoV-2, whereas a 2-fold increase in their infectivity levels relative to Kappa-B.1.617.1 and wild-type pseudoviruses was documented. Other mutations like T478K that is uniquely presented within Delta-B.1.617.2 had no effects on neutralization sensitivity or infectivity relative to wild-type pseudovirus. Interestingly, the combination of L452R/T478K and P681R shown in Delta-B.1.617.2 exhibited enhanced infectivity of 4-fold. Nevertheless, both B.1.617 variants were not as infectious as the Beta-B.1.351 pseudoviruses, which also exhibited reduced neutralization sensitivity (5.4-fold) relative to wild-type or B.1.617 pseudo-variants. In addition, the Delta plus variant (AY.1 or B.1.617.2.1) that includes together with the L452R, E488Q, and P681R also the K417N spike mutation that is exhibited on the spike of Beta displayed high resistance to neutralization by post-vaccination sera, similar to Beta variant of concern. However, Delta plus infectivity levels were similar to those of wild-type. We conclude that both Kappa-B.1.617.1 and Delta-B.1.617.2 variants are neutralized by convalescent and post-vaccination sera, with only a mild decrease in vaccine efficacy against these variants. Still, compared with Kappa-B.1.617.1, Delta-B.1.617.2 variant is more infectious, providing an explanation for its high transmissibility and enhanced worldwide prevalence.

## Results

### Effects of convalescent and post-vaccination sera on the Kappa-B.1.617.1 variant

**Figure 1:**
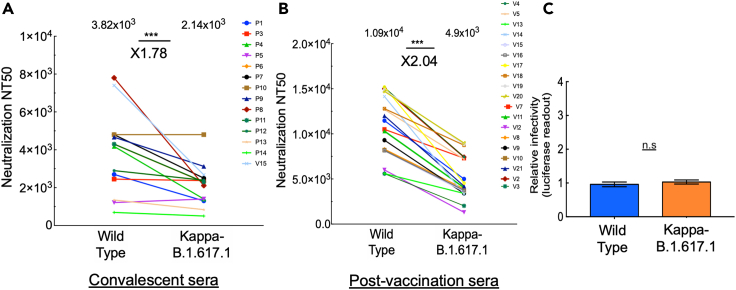
Post-vaccination neutralization sensitivity and infectivity of the Kappa-B.1.617.1 SARS-CoV-2 variant (A and B) Convalescent or post-vaccination sera neutralize Kappa-B.1.6171.1 SARS-CoV-2 pseudoviruses; neutralization assays were performed by transducing HEK-ACE2 cells with pseudoviruses displaying spike protein of either wild-type SARS-CoV-2 or its Kappa-B.1.617.1 variant, in the presence of increasing dilutions of convalescent (A) or post-vaccination (B) sera. At 48 h post-transduction, cells were harvested and their luciferase readings were monitored. Neutralizing potency was calculated at increased serial dilutions, relative to transduced cells with no sera added. Neutralization, NT50, is defined as the inverse dilution that achieved 50% neutralization. Results are the average of two independent biological experiments. Triplicates were performed for each tested serum dilution. Black bars represent geometric mean of NT50 values, indicated at the top. Statistical significance was determined using one-tailed t test; ∗∗∗p<0.001. (C) Infectivity levels of Kappa-B.1.617.1 SARS-CoV-2 pseudoviruses; pseudoviruses bearing wild-type or B.1.617.1 SARS-CoV-2 spike mutations were used to transduce HEK-ACE2 target cells. Equal viral loads were normalized based on their p24 protein levels. At 48 h post-transduction, cells were harvested and their luciferase readouts were monitored. Bar graphs show mean values ±SD error bars of three independent experiments resulted in non-significance statistical difference.

We acquired blood samples from a cohort of convalescent sera (n = 35) drawn from patients who recovered from COVID-19 who presented severe disease symptoms (n = 14) as well as post-vaccination individuals who received two doses (9–11 days post-second dose; n = 19) of the BNT162b2-Pfizer vaccine (see Tables S1 and S2 for additional details on sera samples and timing of collection). All convalescent sera were obtained from patients who were infected with the Alpha-SARS variant. We employed neutralization assays to monitor the potency of each of our sera samples to neutralize pseudoviruses that exhibit spike of wild-type SARS CoV-2, or its Kappa-B.1.617.1 variant. This variant harbors L452R, E484Q, D614G, and P681R spike mutations. Our data showed that both convalescent and post-vaccination sera neutralized B.1.617.1, with only a modest reduction in neutralization potential that was detected in both sera. For convalescent sera, we observed relatively low titers of nAbs, with a 1.78-fold reduction in neutralization sensitivity relative to wild-type SARS-CoV-2 (Figure 1A). Upon vaccination, titers of nAb increased and successfully neutralized B.1.617.1. Herein, a 2.04-fold decrease in neutralization sensitivity of Kappa-B.1.617.1 to post-vaccination sera was observed relative to wild-type SARS-CoV-2 (Figure 1B). We conclude that both convalescent and the BNT162b2 vaccine provide neutralization protection against pseudoviruses that carry the Kappa-B.1.617.1 RBD mutations, with only a moderate reduction of about 2-fold in neutralization potential.

We also tested the infectivity levels of Kappa-B.1.617.1 relative to wild-type SARS-CoV-2. As our platform used single-round pseudoviruses, the term transduction is more suitable than infectivity that would imply the use of SARS-CoV-2 infections. Pseudoviruses were used to transduce HEK-ACE2, and infectivity levels were monitored 48 h post-transduction. Our analysis showed that the infectivity levels of wild-type SARS-CoV-2 and Kappa-B.1.617.1 were similar (Figure 1C). We thus conclude that Kappa-B.1.617.1 spike mutations have no effect on viral infectivity levels.

### Effects of convalescent and post-vaccination sera on the Delta-B.1.617.2 variant

**Figure 2:**
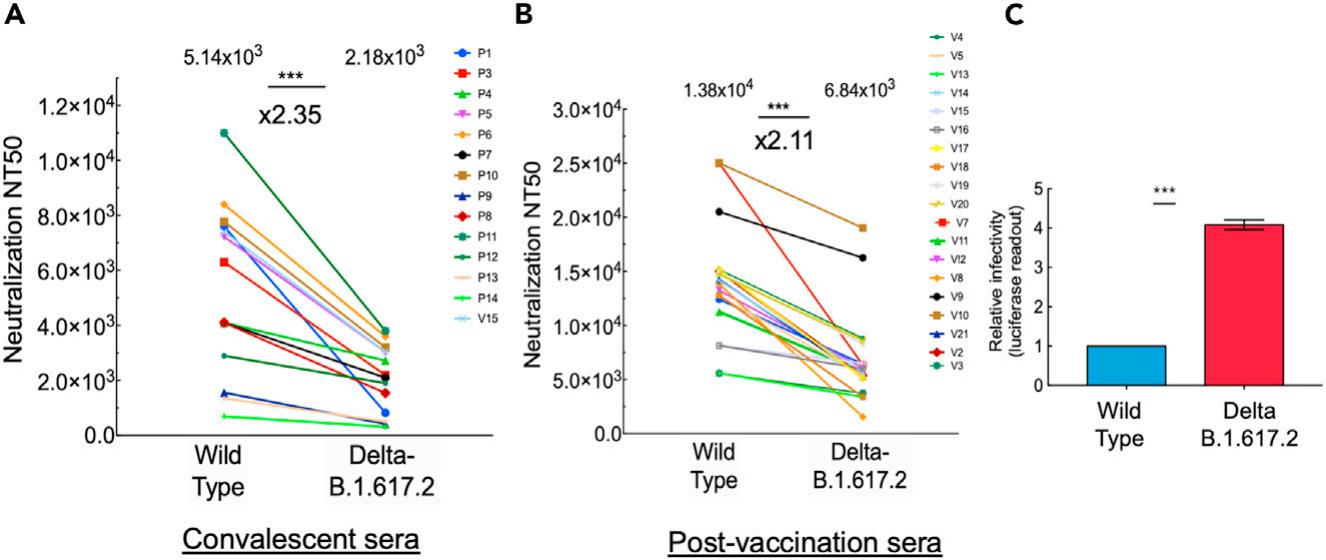
Post-vaccination neutralization sensitivity and infectivity of the Delta-B.1.617.2 SARS-CoV-2 variant (A and B) Convalescent or post-vaccination sera neutralize Delta-B.1.6171.2 SARS-CoV-2 pseudoviruses; neutralization assays were performed by transducing HEK-ACE2 cells with pseudoviruses displaying wild-type SARS-CoV-2 spike or its Delta-B.1.617.2 variant, in the presence of increasing dilutions of convalescent (A) or post-vaccination (B) sera (B). At 48 h post transduction, cells were harvested and their luciferase readings were monitored. Neutralizing potency was calculated at increased serial dilutions, relative to transduced cells with no sera added. Neutralization, NT50, is defined as the inverse dilution that achieved 50% neutralization. Results are the average of two independent biological experiments. Triplicates were performed for each tested serum dilution. Black bars represent geometric mean of NT50 values, indicated at the top. Statistical significance was determined using one-tailed t test; ∗∗∗p<0.001. (C) Infectivity levels of B.1.617 SARS-CoV-2 pseudoviruses; pseudoviruses bearing wild-type, Kappa-B.1.617.1, or Delta-B.1.617.2 SARS-CoV-2 spike mutations were used to transduce HEK-ACE2 cells. Equal viral loads were normalized based on p24 protein levels. At 48 h post-transduction, cells were harvested and their luciferase readouts were monitored. Bar graphs show mean values ±SD error bars of three independent experiments. Measured statistical significance was calculated between experiments by a two-tailed Student's t test; ∗∗∗p ≤ 0.001.

We next employed neutralization assays and monitored the potency of each of our sera samples to neutralize pseudoviruses that exhibit spike of Delta-B.1.617.2 variant. This variant harbors L452R, T478K, D614G, and P681R spike mutations. Our data showed that both convalescent and post-vaccination sera neutralized B.1.617.2, with only a modest reduction in neutralization potential that was detected in both sera. For convalescent sera, we observed relatively low titers of nAbs, with a 2.35-fold reduction in neutralization sensitivity relative to wild-type SARS-CoV-2 (Figure 2A). Upon vaccination, titers of nAb increased and successfully neutralized B.1.617.2. Herein, a 2.11-fold decrease in neutralization sensitivity of Delta-B.1.617.2 to post-vaccination sera was observed relative to wild-type SARS-CoV-2 (Figure 2B). We conclude that both convalescent and the BNT162b2 vaccine provide neutralization protection against pseudoviruses that carry the Delta-B.1.617.2 RBD mutations, with only a moderate reduction of about 2-fold in neutralization potential.

We also tested the infectivity levels of Delta-B.1.617.2, relative to wild-type SARS-CoV-2 and the Kappa-B.1.617.1 variant. Our analysis showed that the infectivity levels of Kappa-B.1.617.1 were similar to those of wild-type SARS-CoV-2. However, Delta-B.1.671.2 exhibited a 4.2-fold increase in its infectivity levels relative to its Kappa lineage or wild-type SARS-CoV-2 (Figure 2C). We thus conclude that Delta-B.1.617.2 variant is more infectious relative to wild-type SARS-CoV-2.

### Single L452R or combined B.1.617 spike mutations affect neutralization sensitivity and infectivity levels against post-vaccination sera

**Figure 3:**
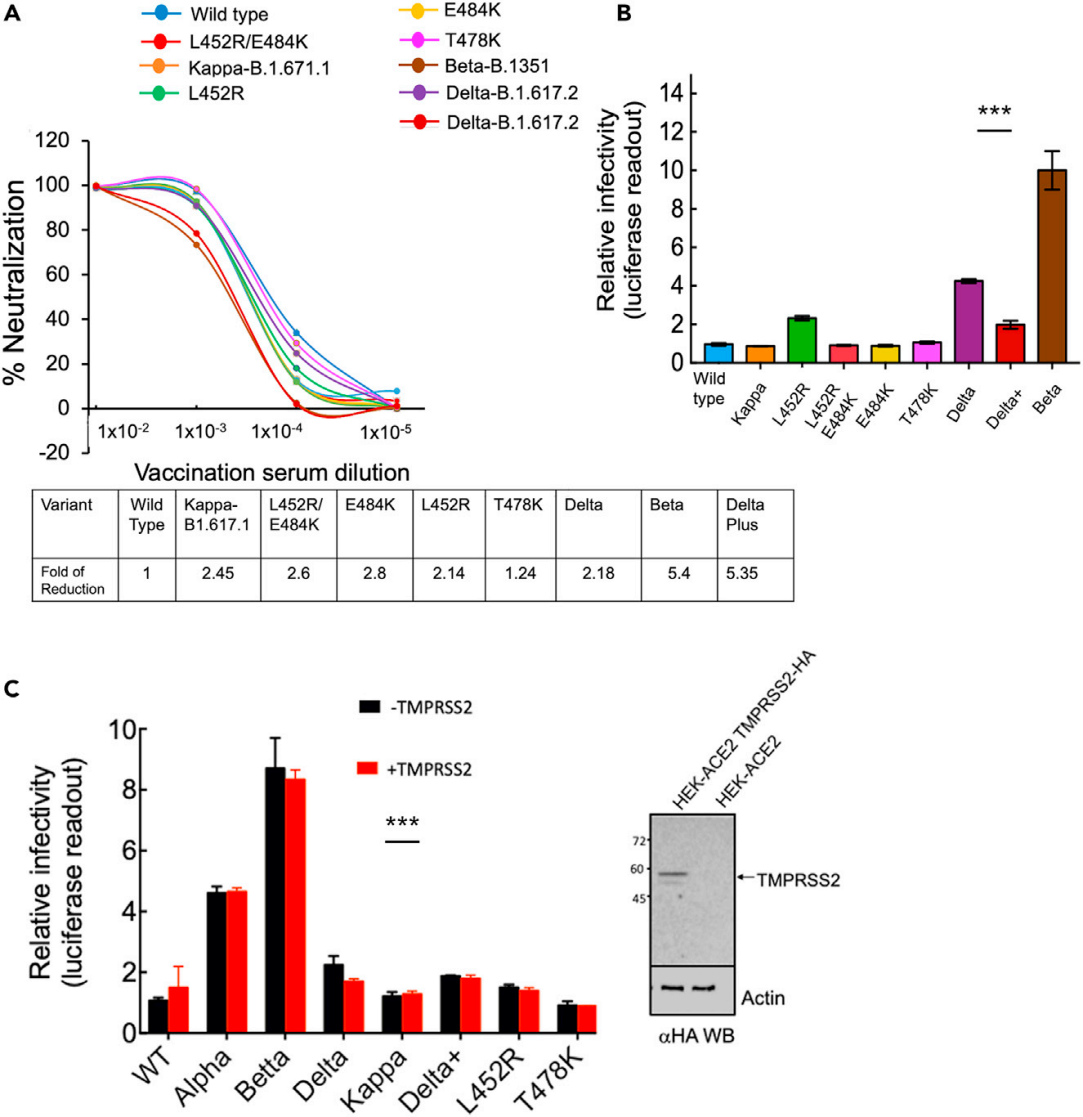
Effects of a single or combined spike mutations presented in B.1.617 pseudoviruses on infectivity and neutralization sensitivity against post-vaccination sera (A) Neutralization potential of pseudoviruses carrying single or combined B.1.617 variants’ spike RBD mutations; neutralization assays were performed by transducing HEK-ACE2 cells with pseudoviruses displaying wild-type SARS-CoV-2 spike or pseudoviruses carrying the single L452R, or combined L452R/E484Q and L452R/E484K RBD mutations, in the presence of increasing dilutions of post-vaccination sera (B). At 48 h post-transduction, cells were harvested and their luciferase readings were monitored. Neutralizing potency was calculated at increased serial dilutions, relative to transduced cells with no sera added. Neutralization, NT50, is defined as the inverse dilution that achieved 50% neutralization. Results are the average of two independent biological experiments. Triplicates were performed for each tested serum dilution. Black bars represent geometric mean of NT50 values, indicated at the top. Statistical significance was determined using one-tailed t test; ∗∗∗p<0.001. The table below summarizes the fold of neutralization decrease relative to wild-type pseudovirus. (B) Infectivity levels of pseudoviruses carrying single or combined B.1.617 variant spike RBD mutations; pseudoviruses bearing wild-type or B.1.617 SARS-CoV-2 spike mutations were used to transduce HEK-ACE2 cells. Equal viral loads were normalized based on p24 protein levels. At 48 h post-transduction, cells were harvested and their luciferase readouts were monitored. Bar graphs show mean values ±SD error bars of three independent experiments. Measured statistical significance was calculated between experiments by a two-tailed Student's t test; ∗∗∗p ≤ 0.001. (C) TMPRSS2 has no effects on viral infectivity of pseudoviruses that infect HEK-293T-ACE2-TMPRSS2. Pseudoviruses bearing the indicated SARS-CoV-2 spike mutations were used to transduce HEK-ACE2 cells and HEK cells that express TMPRSS2. The human protease was transfected into cells 48 h earlier, and its expression was verified in infected cells by western blot. Equal viral loads were normalized based on p24 protein levels. At 48 h post-transduction, cells were harvested, and their luciferase readouts were monitored. Bar graphs show mean values ±SD error bars of three independent experiments. Measured statistical significance was calculated between experiments by a two-tailed Student's t test; ∗∗∗p ≤ 0.001.

As L452R spike mutation is shared by both Kappa-B.1.617.1 and its Delta-B.1.617.2 variants, we were interested in elucidating the role of this mutation in neutralization sensitivity and infectivity of the two variants and the effects of single and combined spike mutations on neutralization sensitivity and infectivity. We thus tested the neutralization potential of post-vaccinated sera toward single or combined RBD mutations that present within the spike of B.1.617 variants. For this, we generated pseudoviruses that carried a single L452R spike mutation, combined L452R/E484Q or L452/E484K RBD spike mutations, as well as single T478K mutants. Our analysis demonstrated that relative to wild-type SARS CoV-2, L452R-pseudoviruses exhibited 2.14-fold reduction in their neutralization sensitivity titers against post-vaccination sera (Figure 3A). Furthermore, combining the L452R with E484Q (i.e., Kappa-B.1.617.1), confirmed the decrease in neutralization sensitivity against post-vaccination sera, presenting 2.45-fold reduction in neutralization sensitivity relative to wild-type SARS CoV-2. Interestingly, switching E484Q with E484K resulted in a slight decrease of 2.6-fold of neutralization sensitivity titers relative to SARS-CoV-2 pseudoviruses. Indeed, E484 is known to serve as a key residue in RBD that promotes neutralization resistance. Thus, pseudoviruses that carried E484K spike mutation exhibited a 2.8-fold decrease in neutralization sensitivity, relative to wild-type pseudoviruses. Similarly, L452/E484K pseudoviruses showed a similar reduction in neutralization sensitivity of 2.6-fold. The Delta-B.1.617.2, which carries T478K mutation, also exhibited a 2.18-fold reduction in its neutralization sensitivity toward post-vaccination sera. Furthermore, single T478K pseudoviruses showed no effects on neutralization sensitivity relative to wild-type or Kappa-B.1.617.1 pseudoviruses. Nevertheless, the decrease of neutralization sensitivity presented by both B.1.617 variants against post-vaccinated sera did not reach the high decrease of post-vaccination sera against the Beta-B.1.351 variant, a 5.4-fold decrease of neutralization sensitivity (Figure 3A). Finally, we also tested neutralization sensitivity of the Delta plus variant and compared it with the other indicated viruses. Surprisingly, our analysis demonstrated that this variant displayed a 5.35-fold increase in its sensitivity to post-vaccination sera, levels similar to those that were observed with Beta variant of concern (Figure 3A).

The infectivity levels of pseudoviruses that carried single L452R, combined L452R/E484Q, or L452/E484K were also determined by transducing HEK-ACE2 (Figure 3B). We showed that pseudoviruses that carried L452R presented enhanced infectivity levels, which were 2.3-fold greater than those of wild-type SARS CoV-2 (Figure 3B). L452R/E484Q (i.e., Kappa-B.1.617.1) exhibited similar infectivity levels as wild-type SARS-CoV-2 pseudoviruses. Interestingly, L452R/E484K also presented similar infectivity levels as wild-type SARS CoV-2 pseudoviruses and E484K single mutation. Finally, Delta-B.1.617.2 variant showed an enhanced 4.2-fold increase in infectivity levels relative to its Kappa-B.1.617.1 variant or wild-type SARS-CoV-2. Interestingly, infectivity levels of the Delta plus variant were similar to those of wild-type SARS CoV-2 pseudoviruses. Pseudoviruses that carried a single T478K spike mutation had no effects on infection levels. Nevertheless, our analysis confirmed the high levels of infectivity of the Beta-B.1.351 variant, 11-fold (Figure 3B) (Garcia-Beltran et al., 2021; Kuzmina et al., 2021).

As most variants also exhibit mutations within their spike FCS, we analyzed the effects of TMPRSS2 host protease on the infectivity levels of our pseudoviruses. Our data show that upon transduction, TMPRSS2 had no effects on infectivity levels, that were similar as obtained without the TMPRSS2 protease (Figure 3C). Western blot and fluorescence-activated cell sorting analyses confirm high level and high frequency of our cells expressing TRMPSS2 (Figures 3C and S1).

## Discussion

**Figure 4:**
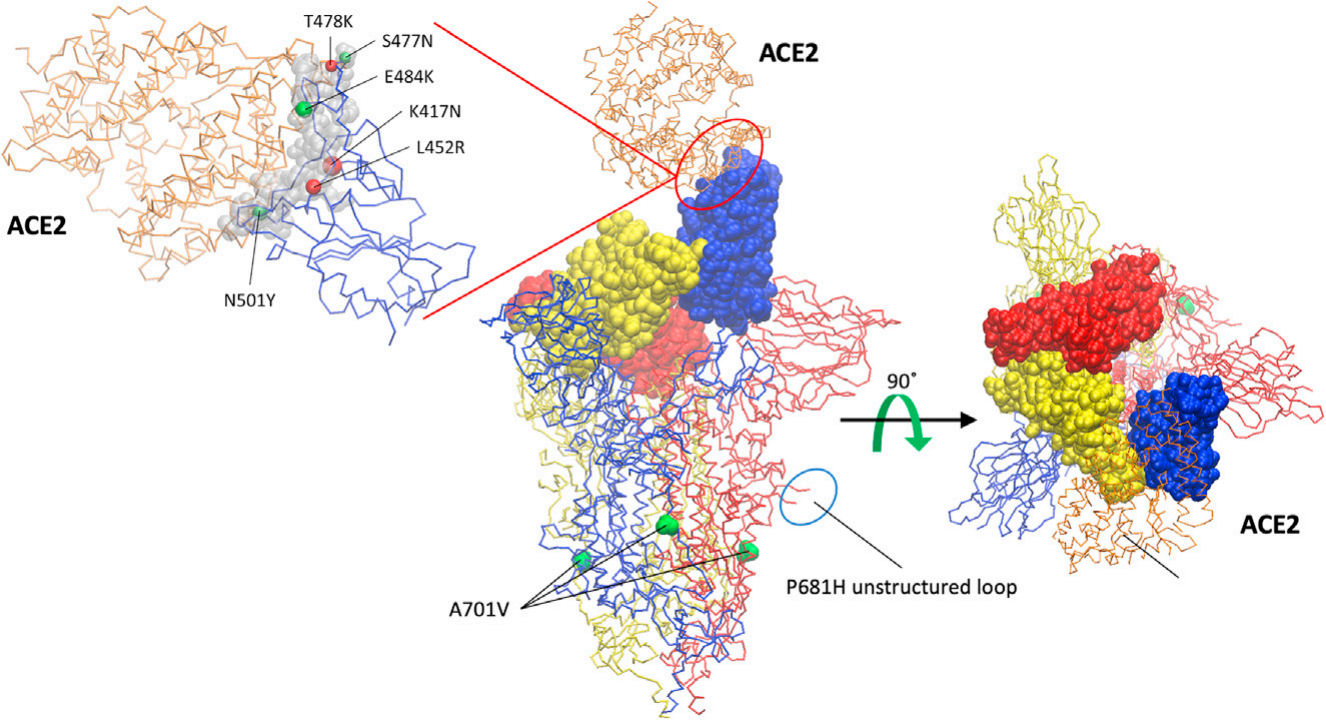
Mutations identified within SARS-CoV-2 variants mapped into the structure of SARS-CoV-2 spike-ACE2 complex The structure of the trimeric spike (S) glycoprotein (PDB: 7df4) (Xu et al., 2021) is shown in a trace backbone representation with individual chains differently colored. ACE2 protein is colored in orange. The RBD domains (amino acid [aa] 333–527) of the spike protein are highlighted using a Van der Waals representation. Mutations S477N, T478K, E484K, K417N, L452R, and N501Y are located in the RBD, but only S477N, E484K, and N501Y form direct contacts with ACE2, as revealed by the RBD/ACE2 interface analysis by using the Knowledge-based FADE and Contacts (KFC2) server (Darnell et al., 2007). Although residues K417 T478, and L452 are not part of the RBD/ACE2 interface, mutations at these positions may affect complex stability indirectly by modifying the properties (conformation) of the nearby interface-forming residues.

The worldwide increase in carriers who are infected with the Delta-B.1.617.2 SARS-CoV-2 variant is of major concern. Most infections occur in young adults and children who have not been vaccinated. Moreover, to some degree, vaccinated individuals are also found to carry the Delta-B.1.617.2 variant, as it rapidly dominates other variants of concern. Nevertheless, despite increase in numbers of infected individuals, disease symptoms are not severe, as cases of hospitalized patients slowly rise. Selective pressure on the virus to adapt its new host led to the early rise of new variants that carry unique mutations within the spike RBD that exhibit high affinity to the ACE2 receptor on human target cells and efficient escape from nAbs (Figure 4). As vaccination programs expand, viral evolution finds ways to the emergence of evolutionarily improved variants that present high transmissibility that promotes reinfection. These variants are defined as a concern by the World Health Organization, as they rapidly spread and potentially compromise vaccine efficiency.

Our analysis confirmed that the B.1.617 variants are successfully neutralized by the BNT162b2 vaccine, with only a mild decrease in neutralization sensitivity. This applied to both convalescent and post-vaccination sera relative to wild-type SARS CoV-2 pseudoviruses. Vaccination successfully induced relatively high nAb titers against both Kappa-B.1.617.1 and Delta-B.1.617.2 variants, increasing the likelihood that it could neutralize B.1.617 variants (Figures 1 and 2). However, infectivity levels of Delta-B.1.617.2 were relatively higher when compared with those of wild-type SARS CoV-2 or its Kappa-B.1.617.1 variant. We argue that enhanced infectivity is the main driving force for the rapid spread of Delta B.1.617.2 variant. Of note, our work used pseudoviruses, which are widely used for SARS-CoV-2 research, and their neutralization titers closely correlate with those measured against live SARS-CoV-2 (Garcia-Beltran et al., 2021; Ju et al., 2020; Kuzmina et al., 2021; Pinto et al., 2020; Yadav et al., 2021).

Furthermore, our results are in partial agreement with recent published work reporting that indeed sera samples drawn 2 or 4 weeks post-second vaccination successfully neutralized both wild-type and B.1.617 variants, with only a modest reduction in relative neutralization sensitivity. However, unlike our data, in this report, the Kappa-B.1.617.1 exhibited slightly higher decrease in its neutralization sensitivity (3.2-fold) relative to the wild-type or its Delta-B.1.617.2 delta variant (which showed similar levels of neutralization as wild-type) (Liu et al., 2021a, 2021b). In our hands, both the B.1.617 variants exhibited about 2- to 2.3-fold decrease in neutralization sensitivity relative to wild-type SARS-CoV-2. However, the Delta-B.1.617.2 variant presented 4-fold higher infectivity levels, relative to wild-type or Kappa-B.1.617.1 variant. In another recent report, B.1.617.1 and B.1617.2 exhibited a slight reduction in neutralization potential relative to the wild-type virus, but not as seen for the B.1.351 virus (Liu et al., 2021a). These results provide an explanation for its rapid transmission and worldwide dominance of the Delta-B.1.617.2 variant. Differences (although minor) between these two reports may be due to the fact that our data come from using pseudoviruses, whereas the others were done with live viruses, measuring plaque assays on Vero cells. Indeed, our pseudovirus system could not recapitulate the effects of FCS mutations that promote cell-cell fusion and formation of syncytia. Recent work has confirmed that the PRRAR-FCS is important for viral transmission, as it provides an advantage for the virus to enter its target cells at or near the cell surface, primarily in lung and in primary human epithelial cells (Johnson et al., 2021; Lau et al., 2020). In such a scenario, the authentic virus avoids the innate IFITM2 response upon entry and supports efficient cell-to-cell transmission (Peacock et al., 2021; Winstone et al., 2021). On the other hand, in viruses that lack the FCS entry is mediated through endosomes and the viruses are exposed to the IFITM2 innate response. In Vero E6 or HEK cells, which do not express TMPRSS2, SARS-CoV-2 in which FCS is deleted gain advantage, potentially due to an increase in spike stability, as spike cleavage may result in premature shedding of the S1 subunit and abrogates receptor binding (Zhang et al., 2020). Thus, knockout of TMPRSS2 or deletion of the FCS resulted in impaired infection, low viral titers that were shed from infected ferret animal model, and reduced transmission to co-housed animals (Peacock et al., 2021). Moreover, the fact that upon propagating the virus in cell culture, FCS sequence is lost or mutated, whereas in infectious viral isolates these mutations are at a very low level further strengthens the assumption that FCS sequence is critical for viral transmission, mainly in clinically relevant human systems (Peacock et al., 2021). In another recent work, the significance of the FCS is further reinforced. FCS-P681R mutation within the Kappa-B.1.617 SARS-CoV-2 variant augments syncytium formation, thus contributing to increased infectivity of the UK and South Africa variants (Ferreira et al., 2021). However, in our study, we could not see such an effect of TMPRSS2 on viral infection (Figure 3C). Another limitation of our pseudo-system is that the infectivity assays employed single-round pseudoviruses and focused only on the early step of spike-receptor binding entry, with no ability to extend the effects on late stages of virus release of infectious particles. Moreover, we did not perform our assays in a clinically relevant system, where viral infection is dependent on TMPRSS2 and requires membrane fusion, thus facilitating syncytium formation. Finally, it may be that the P681H mutation does not impose the same effect as the P681R that appears in B.1.617 variant where studies on the role of P681 on syncytium formation were conducted (Mlcochova et al., 2021). Interestingly, Kappa-B.1.617.1 virus formed smaller plaques than other viruses, pointing to a lower infectivity level relative to other variants (Liu et al., 2021b). In another report by the Landau group, B.1.617 pseudo-variants were also successfully neutralized by convalescent and post-vaccination sera. These authors reported on a 2.3-fold decrease of convalescent sera to neutralize the B.1.617.1 variant, and a ×4 fold decrease in the ability of post-vaccination sera to neutralize the tested pseudoviruses. They also determined that the L452R and the E484Q spike mutation were the key residues for the decreased sensitivity in neutralization potential (Tada et al., 2021). Our results coincide with this conclusion and show that indeed L452R is the key residue in B.1.617 variants that affects neutralization sensitivity and infectivity levels, whereas E484Q or T478K spike mutation has less of an effect on neutralization. However, the combination of L452R and T478K seen in Delta-B.1.617.2 determines its phenotype. Adding the P681R FCS mutation further enhances viral infectivity. Interestingly, switching E484Q with E484K further elevated the resistance of the modified B.1.617/E484K variant, exhibiting 2.6-fold reduction in vaccine neutralization efficacy (Figure 3A). Furthermore, B.1.617.1, or combined L452R/E484Q and L452R/E484K, exhibited similar infectivity levels as wild-type SARS CoV-2 (Figure 3B). In our assays, the decrease in neutralization sensitivity of B.1.617.1 and even the modified B.1.617.1 (L452/E484K) was lower than presented with the Beta-B.1.351 pseudoviruses (5.4-fold reduction in neutralization sensitivity) (Kuzmina et al., 2021) (Figure 3A). Interestingly, the recently identified Delta plus variant displayed in our analysis similar levels of infectivity as the wild-type SARS-CoV-2 pseudovirus, whereas surprisingly it exhibited high resistance to neutralization of post-vaccinated sera, similar to those of the beta variant.

We can thus confirm that the Pfizer vaccine neutralizes the newly emerged B.1.617 variants. Nevertheless, the mild decrease in neutralization sensitivity and primarily the enhanced infectivity of the Delta-B.1.617.2 variant provide an explanation for the rapid spread of this variant and call for measures that will limit viral spread and advance vaccination programs, as its features can harm individuals who are not vaccinated.

### Limitations of the study

There are several limitations of our system that need to be noted. Our work relies on pseudoviruses, which are only used to characterize the first step of the virus life cycle, i.e., binding of the viral particle to the host cell receptor and entry into the target cells that is mediated by the host ACE2 receptor. As documented, we report on the efficacy of the vaccine to neutralize (inhibit viral attachment and entry) B.1.617 variants. We conclude that the spread of the Delta-B.1.617.2 occurs mainly due to its increased infectivity relative to Kappa-B.1.617.1 Furthermore, the pseudovirus system has been broadly used in the literature for testing vaccine neutralization efficiency against circulating variants of SARS CoV-2. Numerous studies have demonstrated by now high correlation between vaccine neutralization titers measured against pseudovirus and live SARS-CoV-2 (Crawford et al., 2020; Garcia-Beltran et al., 2021; Ju et al., 2020; Kuzmina et al., 2021; Pinto et al., 2020; Wang et al., 2020, 2021; Yan et al., 2020). Moreover, the contribution of additional mutations outside of the spike may also affect resistance to neutralization, infectivity levels, or pathogenesis of SARS CoV-2. Additionally, it is worth stating that our findings are relevant only to the tested sera. However, the mid-sized cohort that was analyzed in our work, combined with other reports with similar conclusions, validates our findings.

Our work also implies that in pseudotype SARS-CoV-2 that transduces HEKACE-2 target cells the role of the FCS in the viral spike is non-essential. Thus, we performed our transduction experiments in the presence of TMPRSS2 or in its absence (Figure 3C). According to our data, we could not detect an effect of the TMPRSS2 protease on transduction levels in our hands. We assume that the reason for this is the use of pseudovirus that cannot recapitulate the significant impact of FCS that promotes cell-cell fusion and formation of syncytia. Moreover, HEKACE-2 target cells also do not support fusion steps as efficiently as the authentic target cells of SARS CoV-2 such as Vero cells where plaque and syncytia formation are easily measured.

## STAR★Methods

### Key resources table

**Table:** 

REAGENTS or RESOURCE	SOURCE	IDENTIFIER
**Bacterial and virus strains**		

Competent *E.Coli* DH5α	NEB	Cat#18265017

**Experimental models: cell lines**		

HEK293T cells	ATCC	Cat#3216

**Critical commercial assays**		

T4 ligase	NEB	M0202S
Xba I	NEB	R0145S
Sal I	NEB	R0138S
QuikChange Lightning Site-Directed Mutagenesis kit	Agilent Technologies	#200522
Luciferase assay	Promega	E1500

**Recombinant DNA**		

pCG1-SARS-CoV-2 spike	Hoffmann et al. (2020b)	NA
pLenti-PGK_Luc	Addgene	Cat#19360
pCMV delta 8.2	Addgene	Cat#12263
pCG1_ACE2	Hoffmann et al. (2020b)	NA
pLenti_CMV_PURO	Addgene	Cat#17448
Recombinant proteins	ACROBioSystems	SPD-C82 × 10^9^

**Software**		

Prism 9.0	GraphPad	NA
Photoshop	Acrobat	NA

**Oligonucleotides**		

TTTCAGCCCACATATGGCGTGGGCTAT	Hylabs	FWD-N501Y
ATAGCCCACGCCATATGTGGGCTGAAA	Hylabs	Rev-N501Y
GGACAGACAGGCAACATCGCCGACTAC	Hylabs	FWD-K417N
GTAGTCGGCGATGTTGCCTGTCTGTCC	Hylabs	Rev-K417N
TGTAACGGCGTGAAAGGCTTCAACTGC	Hylabs	FWD-E484K
GCAGTTGAAGCCTTTCACGCCGTTACA	Hylabs	Rev-E484K
ACTACAATTACCGGTACCGGCTGTTC	Hylabs	FWD-L452R
GAACAGCCGGTACCGGTAATTGTAGT	Hylabs	Rev-L452R
GGCCGGCAGCAAACCTTGTAACGGC	Hylabs	FWD-T478K
GCCGTTACAAGGTTTGCTGCCGGCC	Hylabs	Rev-T478K
TATCAGGCCGGCAACACCCCTTGTAAC	Hylabs	FWD-S477N
GTTACAAGGGGTGTTGCCGGCCTGATA	Hylabs	Rev-S477N

### Resource availability

#### Lead contact

Further information and requests for resources and reagents should be directed to the lead contact, and will be fulfilled by the lead contact, Ran Taube (rantaube@bgu.ac.il).

#### Materials availability

All unique DNA constructs, proteins and pseudotyped virus generated in this study are available from the lead contact upon request.

### Experimental model and subject details

#### Human subject collection

The study was conducted in compliance with ethical principles of the Declaration of Helsinki and approved by the Soroka Medical Center Institutional Review Board (protocol 0281-20-SOR). Sera was collected from a 35 cohort of individuals that had COVID19 or were vaccinated with the Pfizer vaccine. Collected sera are summarized in Tables S1 and S2.

#### Bacterial strains and culture

HEK-ACE2 stable cells were cultured at 37°C in a 5% CO2 incubator. Cells were grown in Dulbecco’s Modified Eagle Medium (DMEM) high glucose (Gibco), supplemented with 10% fetal bovine serum (FBS), 2mM GlutaMAX (Gibco) and 100 U/ml penicillin-streptomycin. HEK-ACE2 expressing cells were generated by stable transduction with lentivirus expressing human ACE2. Our pseudoviruses were standardized for equal loads by monitoring p24 levels by ELISA. *E.coli* DH5α bacteria were used for transformation of plasmids coding for lentivirus packaging DNA and SARS CoV-2 spike. A single colony was picked and cultured in LB broth with 50 μg penicillin at 37°C at 200 rpm in a shaker for overnight.

### Methods details

#### Generation of HEK-hACE2 stable cell line

hACE2 (received from S. Pohlmann lab, University Göttingen, Germany) was re-cloned into lentiviral expression vector. Lentiviral particles were produced as described previously (Krasnopolsky et al., 2020) Briefly, HEK293T cells were stably transduced with lentivirus expressing ACE2. Cells were analyzed for hACE2 expression by FACS, using biotinylated-labeled spike (ACROBiosystems). High ACE2 expressing cells were sorted using FACS Aria. ACE2 expression was periodically monitored by FACS. For TMPRSS2 expression, HEK-ACE2 cells were transfected with an expression plasmid of the protease 48hr before infection. Protein expression was verified by western blotting upon harvesting SARS CoV-2 infected cells.

#### Construction of spike mutants

QuikChange Lightening Site-Directed Mutagenesis kit was used to generate amino acid substitutions in the pCDNA spike plasmid (received from S. Pohlmann lab, University Göttingen, Germany), following the manufacturer’s instructions (Agilent Technologies, Inc., Santa Clara, CA). For each mutant the relative oligos that harbored the required mutation were employed.

#### Generation of pseudotyped lentivirus and neutralization assays

Pseudotyped viruses were generated in HEK293T cells. Briefly, LTR-PGK luciferase lentivector was transfected into cells together with other lentiviral packaging plasmids coding for Gag, Pol Tat Rev, and the corresponding wild type or mutate spike envelopes. Transfections were done in a 10 cm format, as previously described and supernatant containing virus were harvested 72hr post transfection, filtered and stored at −80°C (Krasnopolsky et al., 2020). Neutralization assays were performed in a 96 well format, in the presence of pseudotyped viruses that were incubated with increasing dilutions of the tested sera (1:2000; 1:8000: 1;32000: 1:128000) or without sera as a control. Cell-sera were for 1hr. at 37°C, followed by transduction of HEK-ACE2 cells for additional 12 hr. 72hr post transduction, cells were harvested and analyzed for luciferase readouts according to the manufacturer protocol (Promega). Neutralization measurements were performed in triplicates using an automated Tecan liquid handler and readout were used to calculate NT_50_ – 50% inhibitory titers concentration.

#### Pseudoviruses quality control

To determine the titers of pseudoviruses, 100000 ACE2 stable HEK cells were plated in a 12-well plate. 24 h later, decreased serial dilutions of pseudovirus were used to transduce cells. 48 hr. post transduction, cells were harvested and analyzed for their luciferase readouts. p24 ELISA measurements were conducted to ensure equal loads.

### Quantification and statistical analysis

All experiments were in technical duplicates or triplicates. Statistical analyses were performed using GraphPad Prism. Measured statistical significance was calculated between experiments by a two-tailed Student’s t test - P ≤ 0.001. Error bars throughout all figures represent one standard deviation. Specific details on statistical tests and experimental replicates can be found in the figure legends.

## Data Availability

Additional information and data reported in this paper is available from the lead contact upon request.
