# Supplementary material for: SARS CoV-2 Delta variant exhibits enhanced infectivity and a minor decrease in neutralization sensitivity to convalescent or post-vaccination sera
Source: iScience. 2021 Nov 15;24(12):103467. doi: 10.1016/j.isci.2021.103467 (PMC8591850; doi:10.1016/j.isci.2021.103467)
Supplement: Document S1. Figure S1 and Tables S1 and S2 [file mmc1.pdf]

iScience, Volume *24*

Supplemental information

SARS CoV-2 Delta variant exhibits enhanced infectivity and a minor decrease in neutralization sensitivity to convalescent or post-vaccination sera

Alona Kuzmina, Seraj Wattad, Yara Khalaila, Aner Ottolenghi, Benyamin Rosental, Stanislav Engel, Elli Rosenberg, and Ran Taube

Supplementary Figures and Legends

Table 1: Convalescent human sera used in this study (related to STAR)

Serum	Days post symptoms onset	ELISA IgG Liasonx	NT_{50} dilution	Sex/Age	symptoms	Time from disease	Long COVID
P1	63	8	10000	M/45	+++	9 weeks	+
P3	56	37	10000	F/20	+	8 weeks	
P4	21	0	79.36	M/42	++	3 weeks	+
P5	49	6	250	F/35	+	7 weeks	
P6	28	21	833.3	M/32	+	4 weeks	
P7	21	20	10000	F/41	+++	3 weeks	+
P8	63	68	25000	M/67	++++	9 weeks	+
P9	56	23	20000	F/39	++++	8 weeks	+
P10	49	17	10000	M/48	++	7 weeks	+
P11	49	11	833.3	M/48	++	7 weeks	+

Table 2: Post vaccination human sera used in this study (related to STAR)

Serum	Days of collection post dose #1	Days of collection post dose #2	ELISA IgG	NT_{50} dilution	Age/sex	symptoms
V.1.1	21		79	50000	F/27	
V.1.2	21		35	33333.3	F/69	
V.1.3	21		63	33333.3	M/55	
V.1.4	21		109	40000	F/30	
V.1.5	21		119	100000	F/30	
V.2.2		10	259	200000	M/45	
V.2.3		10	254	100000	F/41	
V.2.4		10	190	100000	F/58	
V.2.5		10	156	64000	F/64	
V.2.6		10	400	128000	F/40	
V.2.7		9	223	83333.3	F/54	
V.2.8		12	326	83333.3	F/69	
V.2.9		10	341	83333.3	F/55	
V.2.10		9	211	50000	F/22	
V.2.11		10	222	100000	F/46	
V.2.12		12			M/58	
V.2.13		8			F/43	
V.2.14		36			M/29	
V.2.15		30			F/30	
V.2.16		36			M/56	

V.2.17		34			M/28	
V.2.18		44			M/29	
V.2.19		39			M/25	
V.2.20		34			M/55	
V.2.21		35			F/27	

The study was conducted in compliance with ethical principles of the Declaration of Helsinki and approved by the Soroka Medical Center Institutional Review Board (**protocol 0281-20-SOR**). Sera samples drawn (P samples; n=10) from COVID19 recovered individuals were collected at the indicated time points post onset of disease symptoms and screened by SARS-CoV-2 IgG Liasonx ELISA for specific SARS-CoV-2 IgG. Similarly, sera samples drawn from individuals that were vaccinated with the BNT162b2 Pfizer vaccine were also collected at 3 weeks post first dose (V.1 samples; n=5), or 9 days post the second dose (V.2 samples; n=20). Samples were then monitored by neutralization assays against wild-type or spike mutated SARS-CoV pseudoviruses. (+) symbol represent disease symptoms severity. Long COVID19 are patients that had disease symptoms long after disease onset. No effect of gender and sex was detected. Abbreviation: M - male; F- female

Fig S1: Staining of TRMPSS2 stable cells

Related to Figure 3C

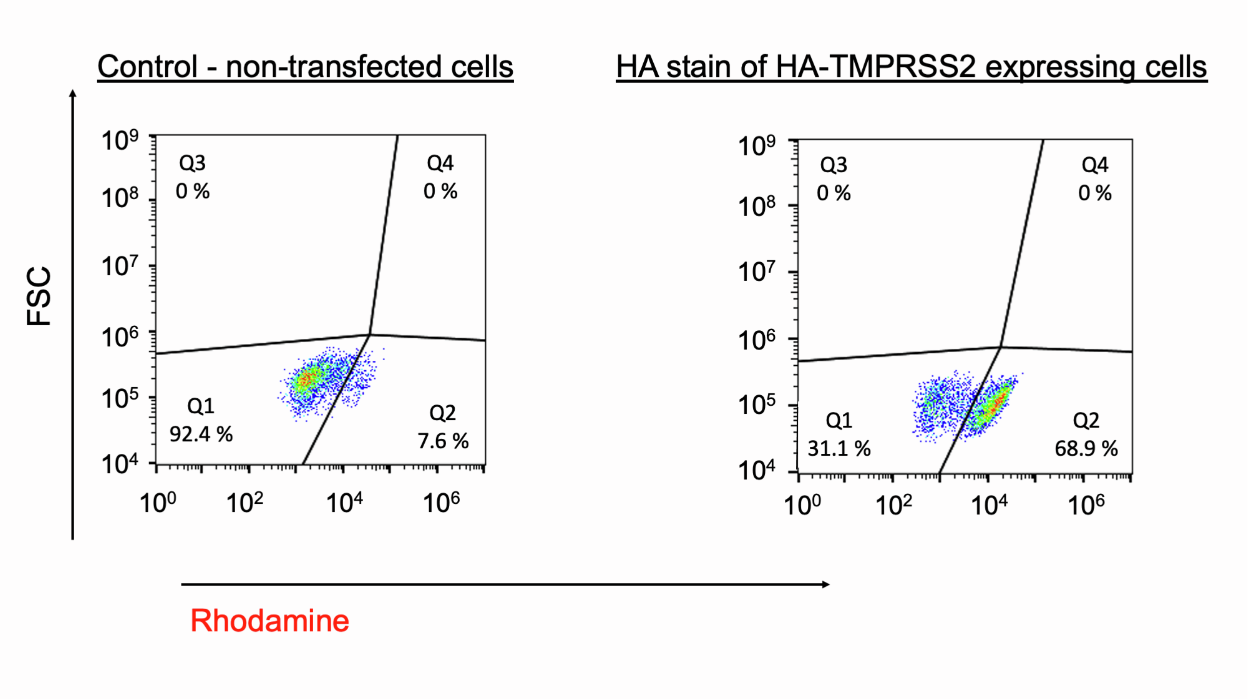

Control HEK-293 non-transfected cells and cells that stably express TMPRSS2-HA were dissociated by EDTA treatment, and washed with PBS-0.01% Tritin washing buffer. Cells were then incubated with 0.04% triton for 10 min on ice, washed and blocked with 4% FCS in PBS. Cells were then incubated with anti-HA IgG (5 µg/ml, Abcam ab9110) for 45 min on ice. After x3 washes with washing buffer, the cells were incubated with Rhodamine-Red labeled goat anti-Rabbit IgG antibody (1:200; Jackson 111-296-003), washed x3 times with washing buffer and analyzed by FACS.
